# Impact of duodenal papilla anatomy on needle knife papillotomy safety and efficacy in patients with difficult biliary canulation

**DOI:** 10.1186/s12893-024-02350-1

**Published:** 2024-02-16

**Authors:** Yunxiao Lyu, Shenjian Ye, Bin Wang

**Affiliations:** Department of Hepatobiliary Surgery, Dongyang People’s Hospital, Affiliated Dongyang Hospital of Wenzhou Medical University, 60 West Wuning Road, Dongyang, 322100 Zhejiang P.R. China

**Keywords:** Needle-knife papillotomy, Papillary anatomy, Biliary cannulation, Adverse events

## Abstract

**Background and aims:**

Needle-knife papillotomy (NKP) is widely performed when biliary cannulation is difficult during endoscopic retrograde cholangiopancreatography (ERCP). However, its safety and efficacy in different types of duodenal papilla are not clear.

**Patients and methods:**

This retrospective study analyzed 217 patients with difficult biliary cannulation who underwent NKP during ERCP procedures from June 2013 to May 2022 in our institution. Patients were classified according to Haraldsson classification type of duodenal papilla: type 1, regular; type 2, small; type 3, protruding or pendulous; and type 4, creased or ridged. Outcome measures were successful biliary cannulation and incidence of adverse events.

**Results:**

Haraldsson classification was type 1 in 115 patients, type 2 in 29, type 3 in 52, and type 4 in 21. Biliary cannulation was successful in 166 patients (76.5%) Success rates according to Haraldsson type were as follows: type 1, 74.8%; type 2, 82.8%; type 3, 80.8%; and type 4, 66.7%. The rates did not significantly differ among the types (*p* = 0.48). Overall incidence of adverse events was 9.22%. Incidence of adverse events did not significantly differ among the types (*p* = 0.69).

**Conclusions:**

NKP was useful to achieve successful cannulation in patients with difficult biliary cannulation. The rate of successful cannulation and incidence of adverse events were similar among the different types of duodenal papilla.

## Introduction

Achieving biliary access is key to performing endoscopic retrograde cholangiopancreatography (ERCP) [1]. However, biliary cannulation is unsuccessful in 5–20% of patients despite continuing improvements in technique and equipment [2, 3]. Several techniques have been recommended when biliary cannulation is difficult [4–7]. Needle-knife papillotomy (NKP) is a commonly used precut technique that is safe and efficacious when performed by experienced endoscopists [8–10]. However, NKP may increase the risk of post-ERCP pancreatitis (PEP), bleeding, and perforation and should be used only in selected patients [11].

Guidelines published by the European Society of Gastrointestinal Endoscopy (ESGE) recommend that NKP be used for individual patients based upon biliary anatomical criteria [12]. However, the supporting evidence for this is low and based on only a few studies [13]. Although previous studies have demonstrated that NKP may be more suitable for small and flat duodenal papilla [14, 15], no randomized controlled trials or prospective cohort studies have yet examined the association of duodenal papilla type with NKP safety and efficacy. Furthermore, there is no current consensus regarding classification of duodenal papillary morphology. This study aimed to compare NKP safety and efficacy between different types of papilla in patients with difficult biliary cannulation (DBC).

## Materials and methods

### Patients

This retrospective study was performed in a tertiary medical center that performs nearly 300 ERCP procedures annually. Institutional ethics committee approval was obtained. Patients who underwent ERCP from June 2013 to May 2022 were initially reviewed. Those with DBC during the procedure who underwent NKP were eligible for study inclusion. Patients with surgically altered anatomy (Billroth-II gastrectomy, Roux-en-Y anastomosis) and those who had undergone a previous ERCP or ERCP for pancreatic indications were excluded. DBC was defined according to the ESGE as the presence of one or more of the following: more than 5 contacts with the papilla whilst attempting to cannulate; more than 5 min spent attempting to cannulate following visualization of the papilla; and more than one unintended pancreatic duct cannulation or opacification [12]. The above three aspects of data were extracted from the ERCP electronic records of our hospital. This electronic record system encompasses comprehensive data, incorporating images of the papilla, the frequency of interactions with the papilla, cannulation time, instances of pancreatic duct entries, intraoperative cholangiographic observations, details on instruments utilized, and more. Patients were classified according to Haraldsson classification type of papilla, which was determined using photography during ERCP: type 1, regular; type 2, small; type 3, protruding or pendulous; and type 4, creased or ridged [16]. Patient data including age, gender, concomitant disease, indication for ERCP, and laboratory findings were recorded from the medical record. Three authors autonomously categorized the duodenal papilla photographs featured in the study. In instances of discrepancies, collaborative discussions were conducted to resolve differences and reach a consensus on the classification of the duodenal papilla types. ERCP was performed by three senior endoscopists using a JF 260 duodenoscope (Olympus, Tokyo, Japan). Selective biliary cannulation was carried out using a Clever Cut sphincterotome (Olympus) and guidewire. NKP was performed using a KD-10-1 needle knife (Olympus) starting from the papillary orifice and proceeding at the 11–12 o’clock position upward over the papillary mound. Continuous pulse oximetry, electrocardiography, and automated blood pressure monitoring was performed during the procedure. Somatostatin was administered to all patients before ERCP to prevent PEP before 2021; after 2021, indomethacin was administered unless the patient had a contraindication.

### Outcome measures

The primary outcome measure was successful biliary cannulation, which was defined as successful introduction of the guidewire into the bile duct. Secondary outcome measures included PEP, bleeding, perforation, and other adverse events as defined by ESGE guidelines [12].

### Statistical analysis

Statistical analyses were performed using SPSS software version 21.0 (IBM, Armonk, NY, USA). Continuous data are expressed as means with standard deviation and were compared using analysis of variance. Categorical data are expressed as numbers with percentage and were compared using the chi-square test. *P* < 0.05 was considered significant.

## Results

### Patient characteristics

**Fig. 1:**
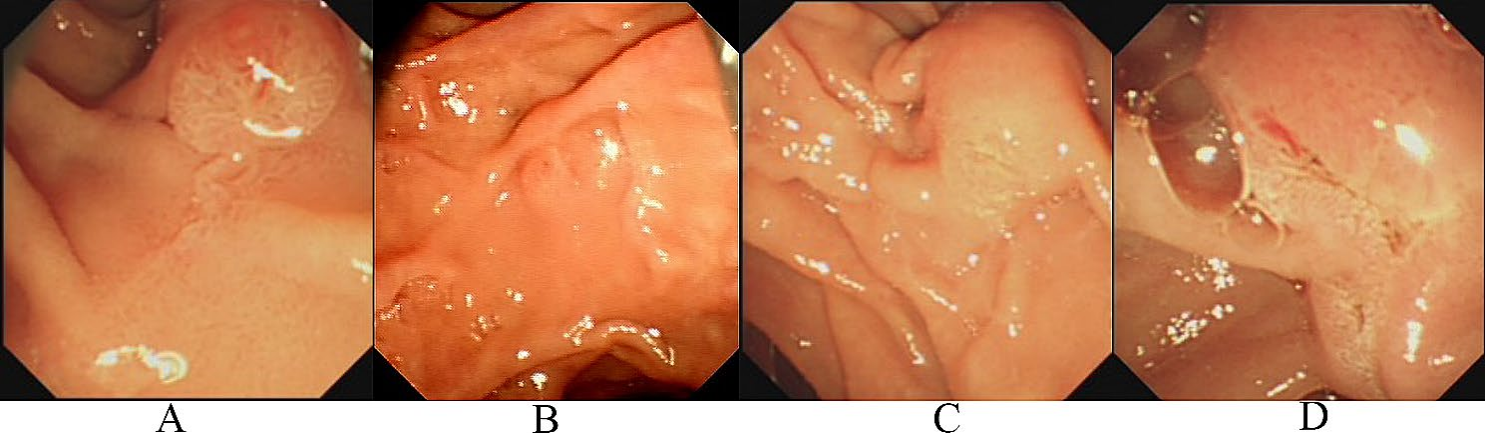
Endoscopic classification of the macroscopic appearance of the Papilla of Vater. **A**: regular papilla; **B**: small papilla; **C**: protruding or pendulous papilla; **D**: creased or ridged papilla

Patient characteristics are summarized in Table 1. Among the 1321 patients who underwent ERCP during the study period, 217 patients met criteria and were included for analysis. One hundred twelve were men (51.61%). Mean age was 63.93 ± 15.40 years. Haraldsson classification was type 1 in 115 patients, type 2 in 29, type 3 in 52, and type 4 in 21 (Fig. 1). Indication for ERCP was common bile duct stones in 177 patients (81.57%), pancreatic neoplasm in 24 (11.06%), cholangiocarcinoma in eight (3.69%), and other in eight (3.69%). Preoperative laboratory data were similar between the four groups. Somatostatin and indomethacin were administered in 98 and 48 patients, respectively; seven patients underwent pancreatic stenting. Method of PEP prophylaxis did not significantly differ among the four groups (Table 2). Eighteen patients (8.3%) had a duodenal diverticulum (Table 2). Papillary balloon dilatation was performed in 47 patients (21.7%). Pancreatic duct cannulation was performed before NKP in 51 patients (23.5%); among these, 39 patients (17.97%) have cannulation < 5 attempts and 12 patients (5.53%) have cannulation ≥ 5 attempts. Pancreatic stent was used in 7 patients (3.23%) (Table 2).

**Table 1:** Characteristics of included patients

Variable	Value
Age, yr	63.93 (15.40)
Male sex	112 (51.61)
Total bilirubin (umol/L)	70.23 (84.11)
Direct bilirubin (umol/L)	50.17 (64.69)
WBC (U/L)	1.31 (3.72)
PLT (U/L)	200.95 (81.91)
ALT (U/L)	134.27 (178.83)
AST U/L)	171.87 (187.38)
Albuminemia (g/L)	38.74 (23.86)
Preoperative CA 19 − 9, U/mL	518.43 (1712.87)
Combines comorbid, n (%)	
Cardiovascular disease	42 (19.36)
Diabetes mellitus	15 (6.91)
COPD	10 (4.61)
MT	6 (2.76)
Others	11 (5.07)
Indication of ERCP, n (%)	
CBD stone	177 (81.57)
Pancreatic neoplasm	24 (11.06)
Cholangiocarcinoma	8 (3.69)
Others	8 (3.69)
Type of Papillary, n (%)	
Type 1	115(52.99)
Type 2	29 (13.36)
Type 3	52 (23.96)
Type 4	21 (9.69)
PEP prophylaxis, n (%)	
Somatostatin	98(45.16)
Pancreatic stents	7 (3.23)
Indomethacin	48(22.12)

WBC, white blood count; PLT, platelet; ALT, alanine aminotransferase; AST, aspartate aminotransferase; COPD, chronic obstructive pulmonary diseases; CBD, common bile duct; Type 1, regular papilla; Type 2, small papilla; Type 3, protruding or pendulous papilla Type 4, creased or ridged papilla

**Table 2:** Details of during the ERCP

Variable	Value
Duodenal diverticulum, n (%)	
Yes	18 (8.29)
No	199 (91.71)
Post-ERCP procedure, n (%)	
EPBD	47 (21.66)
Pancreatic duct cannulation before NKP, n (%)	
Cannulation < 5	39 (17.97)
Cannulation ≥ 5	12 (5.53)
No	166 (76.50)
Pancreatic stent, n (%)	
Yes	8 (3.69)
No	109 (96.31)

EPBD, Endoscopic Papillary Balloon Dilatation; NKP, needle-knife papillotomy

### Successful biliary cannulation

Successful biliary cannulation was achieved in 166 patients overall (76.5%). The success rates based on the Haraldsson types were as follows: type 1, 74.8%; type 2, 82.8%; type 3, 80.8%; and type 4, 66.7%. There was no significant difference in success rates among the types (*p* = 0.48). NKP procedures were performed on 33 patients between June 2013 and December 2017, and on 184 patients between January 2018 and May 2022. There was no statistically significant difference in cannulation success rates between these two time periods (72.73% vs. 77.17%, *p* > 0.05).

### Adverse events

ERCP-related adverse events are shown in Table 3. Overall incidence of adverse events was 9.22%. Incidence of adverse events did not significantly differ among the groups (*p* = 0.69). The most common adverse event was PEP (14 patients, 6.45%). Incidence of PEP did not significantly differ among the groups (*p* = 0.71) (Table 3). Incidence rates of bleeding and cholangitis were similar among the groups. Perforation did not occur.

**Table 3:** Comparison of adverse events of patients after ERCP

	Type of Papillary, n (%)				
	Type 1 (*n* = 115)	Type 2 (*n* = 29)	Type 3 (*n* = 52)	Type 4 (*n* = 21)	*P* value
Adverse events, n (%)	8	3	4	3	0.69
PEP	6	3	3	2	0.71
Bleeding	1	0	1	0	0.79
Perforation	0	0	0	0	-
Cholangitis	1	0	1	1	0.48

PEP: post-ERCP pancreatitis; Type 1, regular papilla; Type 2, small papilla; Type 3, protruding or pendulous papilla; Type 4, creased or ridged papilla

## Discussion

This study compared safety and efficacy of NKP in different types of duodenal papilla in patients with DBC. The rate of successful cannulation and incidence of adverse events were similar between patients with different types of papilla.

Biliary cannulation is the critical first step when performing ERCP. However, cannulation is unsuccessful in up to 20% of patients [1, 2]. Many factors influence the ability to cannulate, including duodenal positioning, papillary anatomy, and operator experience [12, 17]. In 2017, Haraldsson et al. proposed four types of papilla that are cited in the ESGE guidelines [16, 18] and have been validated in several studies [19] because of its substantial inter-and interobserver agreement. Compared with previous studies, this study had similar papilla ratios for the four types. Several studies have discussed the association between cannulation success and papilla morphology [20–22]. Chen et al. reported that cannulation is more difficult and PEP incidence is higher with small papilla and those that protrude or are pendulous [20]. However, there were few studies focused on the papilla morphology and advance canulation techniques. Numerous methods have been proposed to achieve cannulation in difficult cases. The most widely used precutting method is NKP. NKP is highly successful and safe when performed by experienced endoscopists. In our study, we investigated firstly the influence of duodenal papilla morphology on NKP, an advanced cannulation technique. The overall rate of successfully biliary cannulation in our study was 76.50%, which is similar to previously reported rates ranged from 42.2–97% [15]. We found that there was not significantly different among four groups which showed when NKP was effective for all types of papilla. In another study, Canena et al. proposed another classification systems and demonstrated that intradiverticular and diverticular border papilla were the only independent risk factors for difficult rescue needle-knife fistulotomy (NKF) biliary cannulation [19]. However, this classification system is complex, also this study focused on the use of NKF. Several studies have suggested that NKF may be more effective for papilla with a long intramural segment [12, 23]. As we all know, NKP and NKF are different, and the advantages and disadvantages of the two are still controversial [24]. In our center, we mostly use NKP, an advanced technology. A recent study on the morphology and advanced cannulation techniques of the duodenal papilla suggests that precut sphincterotomy (PS) may be more suitable for Type 3 papilla [25]. However, both NKP and NKF were used in this study and PS was performed when pancreatic duct was not accessed or as a salvage technique after failed double guidewire. Although these studies all concern the morphology and cannulation, the methods of papillary classification and advanced cannulation techniques vary among different studies.

Incidence of adverse events did not significantly differ among the papilla types, including incidence of PEP, one of the most serious potential adverse events. Chen et al. showed that small papilla is associated with a higher rate of post-ERCP pancreatitis [20]. A possible explanation is that papillary balloon dilatation was performed more frequently in small papilla. Although a previous study reported that a small papilla was a risk factor for perforation [26], perforation did not occur in our study, possibly owing to its small sample size. However, more high-quality study was required in the future.

This study has several limitations. It was retrospective in design with sample size and procedures were performed by three endoscopists. Therefore, selection bias and operator bias may have been introduced. Secondly, while the incidence of complications in this study aligns with previous research, the limited sample size could potentially influence the outcomes. Therefore, it is imperative to either expand the sample size or undertake high-quality randomized controlled trials for further exploration. In addition, PEP prophylaxis varied among patients, which may have affected the results. Future large-scale prospective studies are warranted to confirm our findings.

In conclusion, NKP was useful to achieve successful cannulation in patients with DBC. Moreover, the rate of successful cannulation and incidence of adverse events were similar among the different types of duodenal papilla.

## Data Availability

The datasets used and/or analyzed during the current study are available from the corresponding author on reasonable request.

## Abbreviations

NKP: Needle-knife papillotomyABP:acute biliary pancreatitis
ERCP: Endoscopic retrograde cholangiopancreatography

## References

[1] Chan CH, Brennan FN, Zimmerman MJ, Ormonde DG, Raftopoulos SC, Yusoff IF (2012). Wire assisted transpancreatic septotomy, needle knife precut or both for difficult biliary access. J Gastroenterol Hepatol.

[2] Wang P, Zhang W, Liu F, Li ZS, Ren X, Fan ZN, Zhang X, Lu NH, Sun WS, Shi RH (2010). Success and complication rates of two precut techniques, transpancreatic sphincterotomy and needle-knife sphincterotomy for bile duct cannulation. J Gastrointest Surg.

[3] Mariani A, Di Leo M, Giardullo N, Giussani A, Marini M, Buffoli F, Cipolletta L, Radaelli F, Ravelli P, Lombardi G (2016). Early precut sphincterotomy for difficult biliary access to reduce post-ERCP pancreatitis: a randomized trial. Endoscopy.

[4] Goldberg E, Titus M, Haluszka O, Darwin P (2005). Pancreatic-duct stent placement facilitates difficult common bile duct cannulation. Gastrointest Endosc.

[5] Fogel EL, Sherman S, Lehman GA (1998). Increased selective biliary cannulation rates in the setting of periampullary diverticula: main pancreatic duct stent placement followed by pre-cut biliary sphincterotomy. Gastrointest Endosc.

[6] Kubota K, Sato T, Kato S, Watanabe S, Hosono K, Kobayashi N, Hisatomi K, Matsuhashi N, Nakajima A (2013). Needle-knife precut papillotomy with a small incision over a pancreatic stent improves the success rate and reduces the complication rate in difficult biliary cannulations. J Hepatobiliary Pancreat Sci.

[7] Katsinelos P, Gkagkalis S, Chatzimavroudis G, Beltsis A, Terzoudis S, Zavos C, Gatopoulou A, Lazaraki G, Vasiliadis T, Kountouras J (2012). Comparison of three types of precut technique to achieve common bile duct cannulation: a retrospective analysis of 274 cases. Dig Dis Sci.

[8] Op den Winkel M, Schirra J, Schulz C, De Toni EN, Steib CJ, Anz D, Mayerle J (2022). Biliary cannulation in Endoscopic Retrograde Cholangiography: how to tackle the difficult papilla. Dig Dis.

[9] Zhang QS, Han B, Xu JH, Gao P, Shen YC (2016). Needle-knife papillotomy and fistulotomy improved the treatment outcome of patients with difficult biliary cannulation. Surg Endosc.

[10] Lim JU, Joo KR, Cha JM, Shin HP, Lee JI, Park JJ, Jeon JW, Kim BS, Joo S (2012). Early use of needle-knife fistulotomy is safe in situations where difficult biliary cannulation is expected. Dig Dis Sci.

[11] Cennamo V, Fuccio L, Zagari RM, Eusebi LH, Ceroni L, Laterza L, Fabbri C, Bazzoli F (2010). Can early precut implementation reduce endoscopic retrograde cholangiopancreatography-related complication risk? Meta-analysis of randomized controlled trials. Endoscopy.

[12] Testoni PA, Mariani A, Aabakken L, Arvanitakis M, Bories E, Costamagna G, Devière J, Dinis-Ribeiro M, Dumonceau JM, Giovannini M (2016). Papillary cannulation and sphincterotomy techniques at ERCP: European Society of Gastrointestinal Endoscopy (ESGE) Clinical Guideline. Endoscopy.

[13] Zhang QS, Xu JH, Dong ZQ, Gao P, Shen YC (2022). Success and Safety of Needle Knife Papillotomy and Fistulotomy based on Papillary anatomy: a prospective controlled trial. Dig Dis Sci.

[14] Katsinelos P, Lazaraki G, Chatzimavroudis G, Zavos C, Kountouras J (2015). The endoscopic morphology of major papillae influences the selected precut technique for biliary access. Gastrointest Endosc.

[15] Kawakami H, Kubota Y, Kawahata S, Kubo K, Kawakubo K, Kuwatani M, Sakamoto N (2016). Transpapillary selective bile duct cannulation technique: review of Japanese randomized controlled trials since 2010 and an overview of clinical results in precut sphincterotomy since 2004. Dig Endosc.

[16] Haraldsson E, Lundell L, Swahn F, Enochsson L, Löhr JM, Arnelo U (2017). Endoscopic classification of the papilla of Vater. Results of an inter- and intraobserver agreement study. United Eur Gastroenterol J.

[17] Gong B, Hao L, Bie L, Sun B, Wang M (2010). Does precut technique improve selective bile duct cannulation or increase post-ERCP pancreatitis rate? A meta-analysis of randomized controlled trials. Surg Endosc.

[18] Testoni PA, Mariani A, Aabakken L, Arvanitakis M, Bories E, Costamagna G, Deviere J, Dinis-Ribeiro M, Dumonceau JM, Giovannini M (2016). Papillary cannulation and sphincterotomy techniques at ERCP: European Society of Gastrointestinal Endoscopy (ESGE) Clinical Guideline. Endoscopy.

[19] Canena J, Lopes L, Fernandes J, Costa P, Arvanitakis M, Koch AD, Poley JW, Jimenez J, Dominguez-Munõz E, Familiari P (2021). Influence of a novel classification of the papilla of Vater on the outcome of needle-knife fistulotomy for biliary cannulation. BMC Gastroenterol.

[20] Chen PH, Tung CF, Peng YC, Yeh HZ, Chang CS, Chen CC (2020). Duodenal major papilla morphology can affect biliary cannulation and complications during ERCP, an observational study. BMC Gastroenterol.

[21] Haraldsson E, Kylänpää L, Grönroos J, Saarela A, Toth E, Qvigstad G, Hult M, Lindström O, Laine S, Karjula H (2019). Macroscopic appearance of the major duodenal papilla influences bile duct cannulation: a prospective multicenter study by the Scandinavian Association for Digestive Endoscopy Study Group for ERCP. Gastrointest Endosc.

[22] Balan GG, Arya M, Catinean A, Sandru V, Moscalu M, Constantinescu G, Trifan A, Stefanescu G, Sfarti CV. Anatomy of Major Duodenal Papilla influences ERCP outcomes and Complication Rates: a single center prospective study. J Clin Med 2020, 9(6). 10.3390/jcm9061637 PMC7356786 32481755

[23] Horiuchi A, Nakayama Y, Kajiyama M, Tanaka N (2007). Effect of precut sphincterotomy on biliary cannulation based on the characteristics of the major duodenal papilla. Clin Gastroenterol Hepatology: Official Clin Pract J Am Gastroenterological Association.

[24] Mavrogiannis C, Liatsos C, Romanos A, Petoumenos C, Nakos A, Karvountzis G (1999). Needle-knife fistulotomy versus needle-knife precut papillotomy for the treatment of common bile duct stones. Gastrointest Endosc.

[25] Angsuwatcharakon P, Thongsuwan C, Ridtitid W, Piyachaturawat P, Kulpatcharapong S, Kongkam P, Rerknimitr R (2023). Morphology of the major duodenal papilla for the selection of advanced cannulation techniques in difficult biliary cannulation. Surg Endosc.

[26] Matsushita M, Uchida K, Nishio A, Takakuwa H, Okazaki K (2008). Small papilla: another risk factor for post-sphincterotomy perforation. Endoscopy.

